# Motor Imagery and Action Observation in Breast Cancer Survivors: Protocol for a Randomized Controlled Trial

**DOI:** 10.2196/85469

**Published:** 2026-03-30

**Authors:** María Pilar Arnal-Vallés, Nelia Soto-Ruiz, Ana Beatriz Bays-Moneo, Cristina García-Vivar, Paula Escalada-Hernández

**Affiliations:** 1 Department of Health Sciences, Public University of Navarre (UPNA), Avda. Barañain S/N., Pamplona, 31008, Spain, +34 948166117; 2 Navarre Institute of Health Research (IdiSNA), Pamplona, Spain

**Keywords:** breast cancer, survivors, survivorship, rehabilitation, motor imagery, action observation, lymphedema, pain, mobility, functionality, strength, upper limb, kinesiophobia, range of motion, imagery ability, randomized, trial, protocol, exercises, oncology

## Abstract

**Background:**

Breast cancer is the most common type of cancer in women worldwide, and its incidence is increasing. Although breast cancer survival is slowly increasing, related sequelae can persist after the disease has been treated. The main physical symptoms associated with breast cancer survival include pain, lymphedema, and associated functional limitations. Although multiple treatments are available for alleviating symptoms in breast cancer survivors, their effectiveness remains limited. Motor imagery (MI) and action observation (AO) therapies, which are based on the theory of motor simulation and are used in multiple fields with satisfactory results, have been proposed as alternatives for treating pain and improving mobility and strength.

**Objective:**

This study aims to design, implement, and evaluate the effectiveness of a program combining MI and AO therapies to improve functionality and mobility and alleviate pain and lymphedema of the affected upper limb in women who have survived breast cancer.

**Methods:**

A randomized controlled clinical study will be conducted in a sample of 108 participants who have experienced breast cancer and, as a result, have pain in the affected extremity, lymphedema, or loss of strength and/or mobility. The intervention group will include 54 participants managed with the MI and AO program (a combination of MI, AO, and mobility exercises), while the control group will consist of 54 women performing mobility exercises alone. Pain intensity, muscle strength, joint range, limb diameter, fear of movement, and imagery capability will be evaluated.

**Results:**

The intervention is expected to yield improvements in pain intensity, joint range, muscle strength, and symptoms associated with lymphedema, among other outcomes. The study was funded in December 2023. The number of participants recruited as of manuscript submission is approximately 80, and data analysis has not yet started. These results will be published in 2026.

**Conclusions:**

The implementation of an intervention based on MI and AO has the potential to positively impact female breast cancer survivors who face physical and psychological sequelae that interfere with their daily lives.

## Introduction

Worldwide, breast cancer accounts for approximately 23.8% of all malignant tumors in women, which establishes it as the most prevalent cancer in this population, with incidence rates continuing to rise [1]. Data from the International Agency for Research on Cancer of the World Health Organization indicate that, in 2022, a total of 2,296,840 new cases of breast cancer were diagnosed among women worldwide [1], with the 5-year global prevalence projected to rise to 8,178,393 cases [1].

The incidence of breast cancer continues to increase, and as mortality decreases, more patients live with the disease for longer, causing the prevalence to also increase [1,2]. According to the GLOBOCAN database, in 2020, the number of deaths worldwide among women with breast cancer was 684,996, representing 15.5% of all deaths from cancer. Improvements in treatments and prevention strategies have allowed for a reduction in mortality, mainly in African countries, but global lifestyle changes have driven an increase in disease prevalence [3]. As a result, the number of breast cancer survivors worldwide is increasing [2], creating significant challenges for long-term follow-up and the management of persistent symptoms [4].

Many breast cancer survivors continue to live with physical and emotional sequelae as a result of the treatments received and the oncological process itself. These late and persistent side effects not only compromise quality of life and functionality but may also prevent survivors from integrating the cancer experience into their daily lives [5]. Among the most common physical sequelae are pain, lymphedema, and postmastectomy syndrome. The latter is characterized by a burning sensation, tightness, stabbing, and pain in the chest, armpit, upper medial part of the arm, and surgical scar, characteristics similar to those of phantom limb syndrome. The likelihood of developing lymphedema is greater in women undergoing axillary lymphadenectomy, sentinel node biopsy, or mastectomy [6]. Lymphedema causes a decrease in the functionality of the affected arm, negatively impacting quality of life. This condition is estimated to affect 35% of patients and is associated with pain and functional limitations in activities of daily living (ADLs) [7].

Although it is one of the most frequent symptoms, pain secondary to breast cancer has not been sufficiently investigated [8]. It has been ascribed to a number of etiologies, such as cervical radiculopathy, rotator cuff tendinopathy, postmastectomy syndromes, or neuropathies, as well as preexisting comorbidities (back pain, arthritis, osteoarthritis, and axillary lymphadenectomy accompanied by chemotherapy and/or radiotherapy), young age (18-39 years), fibromyalgia, and depression or anxiety about the possibility of experiencing chronic pain [9]. There is existing evidence suggesting that breast cancer is associated with pain that can become chronic, affecting both patients and survivors.

Various interventions have been developed in an attempt to improve functionality and indirectly reduce pain [10]. Nonpharmacological strategies used in physiotherapy include multicomponent training (aerobic exercise, flexibility, and strength), neuromuscular bandaging, manual lymphatic drainage, and multimodal therapy. However, in many cases, the pain persists after the therapies have been completed [11].

In light of this situation, it is necessary to assess the effectiveness of emerging therapies, such as motor imagery (MI) and action observation (AO). Both interventions are based on simulation theory, which suggests that imagined or observed actions activate brain regions similar to those involved in the actual execution of the movement [12]. Specifically, MI is based on the fact that imagining the performance of an action stimulates the same parts of the brain map involved in performing the action itself [12]. This therapy has proven useful in various fields, such as sports, psychology, rehabilitation, medicine, and education [13]. Studies have reported beneficial effects of MI on the range of motion, strength, pain, and functionality of the affected limb [14-20]. For example, in the case of phantom limb syndrome, patients have reported a significant reduction in pain, which even disappeared in some cases [15]. There is recent randomized controlled trial evidence suggesting that MI-based interventions can increase physical activity levels and self-efficacy in women with breast cancer, supporting the potential of MI as a behavioral and rehabilitation strategy [21].

AO, meanwhile, is based on the hypothesis that, when observing the performance of an action or movement in the first or third person, the neural circuits involved in the execution of the action are activated [12]. This mechanism has been closely linked to the mirror neuron system, which responds during both execution and AO, providing a neurophysiological substrate for observational learning [22,23]. In other words, observing an action engages brain processes similar to those used when performing the action itself [12]. Similar to MI, AO has been applied in different disciplines and has shown positive results in the reorganization of action, motor control, and functional rehabilitation [24].

Individually, both MI and AO have demonstrated effectiveness in improving muscle strength, joint range, pain intensity, and functionality [15,19,24]. However, there is current evidence suggesting that their combined use produces greater activation of motor brain regions than when applied individually [25], although studies assessing the effectiveness of this combined therapy remain scarce [26]. A systematic review and meta-analysis published in 2025 has reported that combining both therapies effectively improves motor functionality, gait speed, upper-extremity functionality, pain intensity, and fear of movement, especially in the upper extremities [27]. Despite their promising potential, the application of these techniques within oncology—and especially among breast cancer survivors—has been insufficiently investigated, leaving a significant gap in the evidence base.

Therefore, the aim of this project is to design, implement, and evaluate the effectiveness of a program combining MI and AO therapies to improve functionality and mobility and alleviate pain and lymphedema of the affected upper limb in women who have survived breast cancer.

## Methods

### Design

A randomized controlled trial will be conducted to test the following hypothesis: a combination of MI and AO therapies is effective for improving mobility and alleviating pain and lymphedema of the upper limb in breast cancer survivors.

The protocol follows the SPIRIT (Standard Protocol Items: Recommendations for Interventional Trials) guidelines [28] and has been registered on ClinicalTrials.gov (NCT07067710). Future publications of the study results will be reported in accordance with the CONSORT (Consolidated Standards of Reporting Trials) guidelines [29].

### Setting

The study will be carried out in Navarra, an autonomous community and province in northern Spain. Information about the study will be disseminated through social networks and newspapers, and collaboration will be requested from SARAY, the Navarra Breast Cancer Association, a nonprofit regional association that promotes comprehensive care for women who have experienced breast cancer and provides biopsychosocial support through different services (eg, psychology, physiotherapy, and accompaniment).

### Study Participants and Eligibility Criteria

Female survivors of breast cancer who reside in Navarra will be invited to participate in the study. The inclusion criteria for participants in the study will be being female, being aged over 18 years, having a diagnosis of breast cancer, having completed any active cancer treatments (chemotherapy, radiotherapy, and/or immunotherapy), and being disease free at the time of data collection. Women undergoing hormonal therapy after treatment completion will also be included as hormonal treatment is not considered an active cancer therapy and is commonly prescribed among breast cancer survivors. Conversely, participants will be excluded if they have a diagnosis of cancer other than breast cancer or evidence of recurrence or metastasis requiring new treatment or are undergoing active cancer therapy (for recurrent or newly diagnosed disease).

### Sample Size

To determine the sample size, we assumed a type I error of 0.05 and a type II error of 0.20 (80% power). The calculation was based on a minimum detectable difference of 1.5 points in pain intensity measured using the numerical rating scale (NRS). This minimum difference aligns with established minimal clinically important difference values for the NRS in breast cancer–related pain contexts, which range from 1.39 to 2.0 points [30-33].

An estimated SD of 2.61 was selected based on a conservative approach considering the variability reported in breast cancer populations using the NRS, where SDs range from 1.4 to 2.8 [34-37]. This conservative estimate ensures adequate statistical power even if data dispersion is greater than anticipated. Considering an estimated 10% dropout, the required sample size was 108 women, with 54 allocated to the control group (CG) and 54 allocated to the intervention group (IG). The power calculations were performed using the GRANMO online program (version 7.12; Programa de Registre Gironí del Cor).

### Randomization, Allocation, and Blinding

The participants in the study will be randomly assigned to 1 of 2 groups: the IG (MI+AO+mobility exercises) or the CG (mobility exercises alone). All the participants will perform mobility exercises of the upper limb involving flexion, extension, abduction, adduction, internal rotation, and external rotation for approximately 20 minutes between 1 and 3 times a day 3 times a week for the duration of the intervention (6 weeks).

The randomization process will be carried out using the Research Randomizer tool and an allocation ratio of 1:1 with the aim of guaranteeing impartiality and validity in the study and ensuring a balanced distribution, simple randomization, and a reduction in biases while preserving the integrity and reliability of the results. The randomization list will be created by a member of the research team without contact with participants or access to personal or clinical information.

This is a single-blind study, as neither participants nor the researchers delivering the intervention can be blinded. Blinding will be maintained for the researchers responsible for collecting outcome data, administering evaluation instruments, and performing the statistical analyses.

### Intervention: the IM-OA23 Program

The study intervention is part of the IM-OA23 program, a structured initiative designed to integrate MI and AO into rehabilitation strategies. Support for the intervention will be presented in video format and will be accompanied by instructions for performing the MI and OA therapies as well as the additional mobility exercises, with a video prepared to help participants perform the therapies.

**Figure 1.**
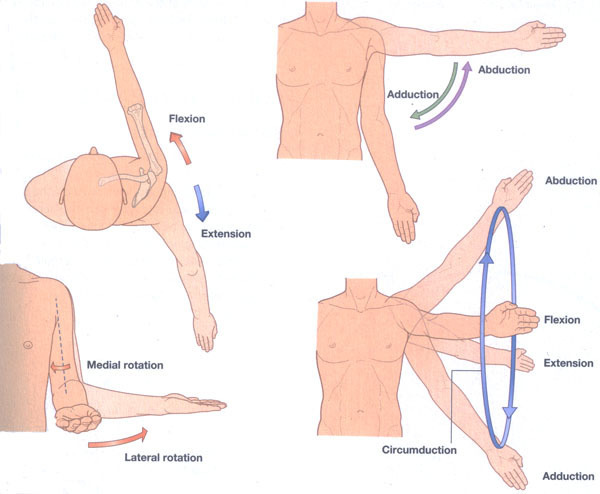
Anatomical movements performed [38].

The intervention will last 6 weeks, during which the content of the program will be progressively modified. During the first 2 weeks, the following anatomical movements will be visualized and imagined, with an approximate duration of 20 seconds per movement: (1) arm flexion from 0 to 180°, (2) arm extension from 0 to −10°, (3) arm abduction from 0 to 180°, (4) arm adduction from 0 to 45°, (5) internal arm rotation from 0 to 45° with the elbow flexed at 90°, (6) external arm rotation from 0 to 45° with the elbow flexed at 90°, and (7) forward and backward arm circumductions (Figures1  2 [38]). In the third and fourth weeks, the participants will progress to performing the anatomical movements described above with a small weight, for example, a 1-kg dumbbell. The videos will depict a woman dressed in black in the frontal and lateral planes performing the described anatomical movements. In the final 2 weeks (fifth and sixth), the content will focus on ADLs through the following tasks: (1) reaching for a jar, (2) raising and lowering blinds, (3) drawing a curtain, (4) changing a light bulb, (5) stirring a pot, (6) scratching one’s back, (7) stretching, (8) putting on and taking off a coat, (9) hugging someone, (10) cleaning a window pane, (11) turning the steering wheel of a car, and (12) throwing a ball.

**Figure 2.**
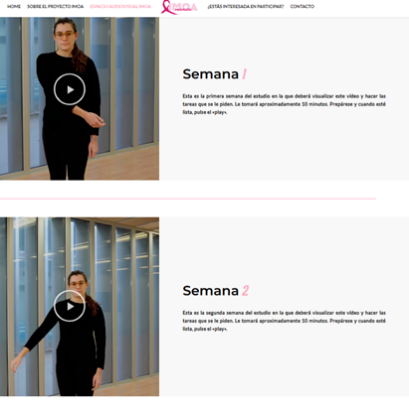
Audiovisual resources developed. The images illustrate the exercises assigned for weeks 1 and 2.

For each of the movements and ADLs, AO will be performed first, followed by MI. After the completion of the MI and AO blocks, the participants will also carry out mobility exercises involving repeating the movements they saw in the videos.

As indicated, all the participants will perform upper-extremity mobility exercises involving flexion, extension, abduction, adduction, internal rotation, and external rotation. They will perform the mobility exercises for approximately 20 minutes per session between 1 and 3 times per day on 3 different days per week throughout the 6-week intervention period. The minimum dose will be 1 time per day on 3 different days per week, and the maximum dose will be 3 times per week on 3 different days per week.

**Figure 3.**
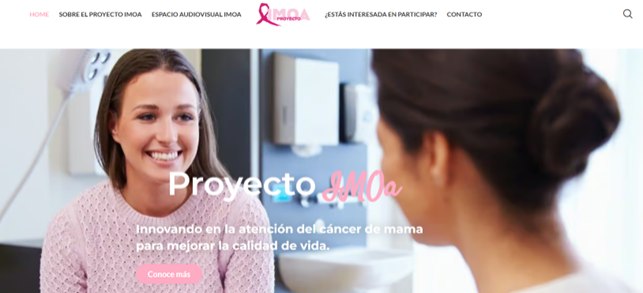
Web page created for the IM-OA23 project. The image shows the project name accompanied by the following phrase: “Innovating in breast cancer care to improve quality of life. Learn more.”

Each participant will be instructed on how to access the audiovisual resources developed specifically for this study and hosted on a dedicated website (Figure 3). They will also receive an information sheet describing the exercises and a field diary to record the frequency of intervention practice during the established weeks. At the end of the explanation, the participant will be scheduled for data collection at 6 weeks (T1) and 3 months (T2).

The videos will be structured as follows: first, a black screen will appear, and a female voice will provide the instructions for performing the therapies. Next, the video will display the movement chosen for the AO therapy. The black screen will then appear again, and 2 acoustic signals will play, indicating the start and end of the MI task. During the MI phase, the participants will be instructed to perform kinesthetic MI from a first-person perspective, imagining themselves executing the observed movement and focusing on the sensations associated with the action. The same process will be subsequently repeated with the next selected movement. The videos will last between 6 and 10 minutes. After viewing the videos and performing the MI and AO therapies, the participants will actively perform the exercises displayed previously in the screens.

### Control Condition

The CG will perform the same movement exercises as the IG but without the addition of MI or AO therapies. All the participants will perform mobility exercises of the upper limb involving flexion, extension, abduction, adduction, internal rotation, and external rotation for approximately 20 minutes 3 times a day 3 times a week for the duration of the intervention (6 weeks).

Once participants consent and are allocated to the CG, sociodemographic and clinical data will be collected, and the remaining variables will be measured (T0). Finally, participants will receive detailed instructions on the exercises, including the number of series and repetitions per series, along with a diary to record exercise frequency throughout the established 6 weeks. Follow-up assessments will be scheduled at 6 weeks (T1) and 3 months (T2).

### Variables and Measurement Instruments

The independent variable will be the IM-OA23 program, which will combine MI and AO therapies. There will be 6 dependent variables.

Pain is the primary outcome variable. It will be evaluated using the NRS, one of the most widely recommended and commonly used tools for assessing pain intensity in a simple, quick, and reliable way. It is an objective scale that quantifies the intensity of pain, similar to the visual analog scale. The NRS is an 11-point scale presented as a horizontal line, with 0 indicating “no pain” and 10 indicating “the worst pain imaginable” or “the worst pain that can exist.” Participants will be asked to report their average pain or pain experienced in the previous 24 hours [39].

Joint range, the degree of movement of the shoulder joint, will be measured using a goniometer. Flexion, abduction, and internal and external rotation will be measured using previously established reference points [40].

Muscular strength, the grip strength of the affected limb, will be measured using a dynamometer (Jamar). The measurement will be made with the participant in a sitting position with the elbow at 90° flexion, the shoulder close to the torso, and a neutral wrist position. The maximum achievable force will be recorded from the best value of 3 attempts [41,42].

Fear of movement will be measured with the Tampa Scale of Kinesiophobia, adapted to Spanish. The scale consists of 11 items rated on a 4-point Likert scale (1-4), yielding a total score from 11 to 44, with higher scores indicating greater fear of movement [43,44].

The diameter of the affected limb will be measured using anthropometric tape with a precision of 1 mm. The measurement will be made at the midpoint between the acromion and the olecranon, leaving the tape perpendicular to the extremity without compressing the area. The midpoint will be located at 90° elbow flexion, and subsequently, the measurement will be obtained with the elbow in extension; the result will be recorded in centimeters [45].

Imagination capability will be assessed using the Movement Imagery Questionnaire–Revised, Second Edition, an 8-item instrument. The questionnaire requires 2 tasks: first, creating a drawing or a mental image of a movement and, second, attempting to feel the sensation of performing the movement without physically executing it. The difficulty of each item will be rated on a 7-point scale ranging from 1 (“very difficult to see/feel”) to 7 (“very easy to see/feel”). The total score is obtained by adding the scores of all the questions. Higher scores correspond to greater ease of imagination and perceived vividness [46].

### Data Collection

As previously described in the Setting section, participants will be enrolled through the dissemination of study information via various media, including the press, social networks, and SARAY. The study information will include a Google Forms questionnaire in which participants can express their interest in taking part and provide their contact information. All respondents to the questionnaire will be invited to an individual meeting with the research team, during which study information will be provided, questions will be addressed, and written informed consent will be obtained upon confirmation of participation. Subsequently, the planning of the intervention and data collection will be explained, and the assigned group will be indicated to each participant.

**Figure 4.**
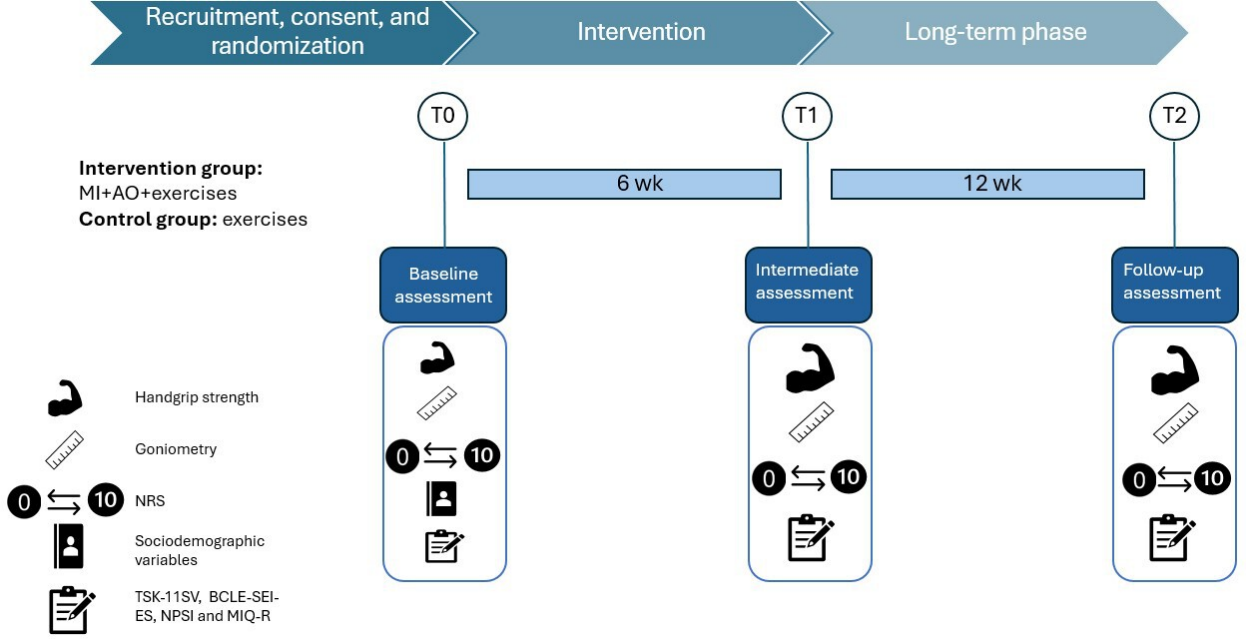
Timeline of the study. AO: action observation; BCLE-SEI-ES: Spanish version of the Breast Cancer and Lymphedema Symptom Experience Index; MI: motor imagery; MIQ-R: Movement Imagery Questionnaire–Revised; NPSI: Neuropathic Pain Symptom Inventory; NRS: numerical rating scale; TSK-11SV: Spanish version of the Tampa Scale of Kinesiophobia.

In both the IG and CG, outcomes will be assessed at 3 time points. The preintervention time point (T0) will coincide with the initial meeting and study inclusion, during which sociodemographic and clinical data will be collected, along with the other study variables. The postintervention time point will take place at 6 weeks (T1). Follow-up (T2) will subsequently be carried out 3 months after the end of the intervention to determine whether the effects have persisted over time (Figure 4). Pain will be considered the primary outcome, and the primary time point to evaluate the effectiveness will be T1. To collect the data, an electronic questionnaire will be designed with the help of the Microsoft Forms tool.

The IG will participate in the IM-OA23 program.

### Data Analysis

First, a descriptive analysis of all study variables will be performed. Continuous variables will be summarized using means and SDs (or medians and IQRs), and categorical variables will be summarized using frequencies and percentages.

The normality of outcome distributions will be assessed for each study group and time using the Kolmogorov-Smirnov test with Lilliefors correction. If normality assumptions are not satisfied, equivalent nonparametric approaches will be considered.

Baseline comparability between groups will be assessed using a 2-tailed Student *t* test (or Wilcoxon rank-sum test if normality is not met) for continuous variables and chi-square or Fisher exact tests for categorical variables.

Second, inferential analyses will be carried out following an intention-to-treat approach whereby all randomized participants will be included in the analyses focused on between-group comparisons (intervention vs control) based on pretest-posttest change scores. Missing data will be imputed and included in all analyses.

The primary analysis will consist of evaluating the effect of the intervention on pain at T0, T1, and T2 comparing changes between the IG and CG from baseline using a linear mixed-effects model.

For all outcome variables meeting normality assumptions, longitudinal trajectories will be evaluated using linear mixed-effects models with participant-specific random intercepts for within-subject correlation. The models will include fixed effects for group (control vs intervention), time (baseline, T1, and T2), and the group × time interaction. Standardized effect sizes (Cohen *d*) will be calculated from the adjusted between-group differences in change from baseline estimated by the mixed-effects models. Effect size will be interpreted as very small for values of less than 0.20, small for values of 0.20 to 0.49, moderate for values of 0.50 to 0.79, and large for values of 0.80 or more.

For variables not meeting normality assumptions, appropriate nonparametric methods will be considered. Between-group comparisons of change scores at each follow-up will be performed using the Wilcoxon rank-sum test.

In all cases, the significance level will be set at .05. The data will be analyzed using the SPSS program (version 27; IBM Corp).

### Ethical Considerations

This project has been approved by the Ethics Committee for Drug Research of Navarra (PI_2023/81), and the necessary institutional authorization to carry out the study has been obtained . Participants will receive information about the project, their degree of involvement, and the legal requirements we will follow in an information sheet, as well as an informed consent form to be signed if they agree to voluntarily participate. Participants will not receive any compensation for participating in the study. The study will exhaustively comply with all the ethical-legal requirements in force related to research involving humans [47]. In order to maintain privacy and confidentiality, all data will be obtained under a personal and unique code. In the event that the IM-OA23 program is shown to be effective in improving pain, mobility, and lymphedema in breast cancer survivors, once the research is completed, breast cancer survivors from the CG will be invited to participate in the full program, and the resources used for the IG will be provided. In addition, the feasibility of incorporating the tool into the national health system will be assessed, with the aim of making it accessible to the entire population.

## Results

The intervention is expected to yield improvements in pain intensity, joint range, muscle strength, and symptoms associated with lymphedema, among other outcomes. The study was funded in December 2023. The number of participants recruited as of manuscript submission is approximately 80, and data analysis has not yet started. Data collection is expected to be finished by June 2026, and the results will be published in 2026.

## Discussion

### Expected Findings

This randomized controlled trial will investigate the potential effect of the combination of MI and AO therapies in improving the functionality of the affected upper limb in breast cancer survivors. Although previous studies have demonstrated the effectiveness of combining these therapies in improving pain, fear of movement, and other outcomes in conditions such as phantom limb syndrome, stroke, or chronic pain, no studies to date have examined their impact in female breast cancer survivors with upper-extremity symptoms. This study seeks to address that gap. Given the similarities of symptoms in breast cancer survivors to those observed in other conditions for which MI and AO therapies have proven effective, the potential benefits for quality of life and treatment appear promising.

### Implications for Clinical Practice

The results of this study have the potential to generate evidence supporting new therapeutic approaches for addressing functional limitations and mobility problems of the affected limb in women with breast cancer—a population in which these symptoms are common and frequently insufficiently treated.

This project is of particular interest given the growing number of female breast cancer survivors, many of whom face limitations in recovery and reintegration into daily activities due to disease-related consequences.

The implementation of the IM-OA23 program could directly benefit female breast cancer survivors with upper-limb sequelae. Aligned with current priorities for survivorship care outlined in the Strategic Framework of Primary and Community Care and the Comprehensive Plan of Care for Long-Term Cancer Survivors of the Spanish Society of Medical Oncology [48], the IM-OA23 program also represents an innovative approach with potential for significant social and economic impact nationally and internationally.

Short-term benefits may include improved pain management, increased mobility, and reduced lymphedema. If effective, the program could offer breast cancer survivors additional tools to alleviate pain and lymphedema while improving upper-limb mobility. This could reduce the need for pain medication and improve the ability to perform daily activities such as dressing, grooming, and reaching for objects while also helping prevent complications such as lymphedema-related infections.

Evaluating the medium-term impacts of the program would generate valuable knowledge on the translation of research into health care practice. Such assessments could clarify the program’s sustainability, its integration into clinical workflows, and its potential to inform evidence-based guidelines for survivorship care.

In the long term, the results of this study could have a significant impact on alleviating symptoms affecting the lives of breast cancer survivors. At the same time, it may contribute to improving the quality and continuity of survivorship care while offering a cost-effective tool to improve patient management and expand the resources available to health care professionals.

In addition, if effective, the program could be proposed as an open access resource at the international level—adapted into different languages—to be accessible not only to rehabilitation professionals but also directly to patients. Such an approach would facilitate global reach, allowing for its use from anywhere while enabling the monitoring of symptom evolution and adherence to the intervention and the timely resolution of emerging issues.

Finally, this study highlights the need to prioritize research on women and the health challenges they face. Beyond its immediate contributions, it has the potential to open new avenues for future research aimed at understanding and addressing symptoms that emerge with the chronification of specific pathologies.

### Study Limitations

This study has several limitations. First, the follow-up period of the intervention is short, restricted to 3 months. At this time point, the long-term effects of the intervention may not yet have fully emerged, potentially underestimating its sustained impact. However, it should be noted that the selected follow-up times are based on previous studies analyzing the combination of MI and AO therapies. It should also be noted that follow-up is constrained by the funding period and by the practical and organizational limitations of participants. Therefore, future research should include long-term evaluations of the intervention’s outcomes.

Second, given the behavioral nature of the program, it will not be possible to blind either the participants or the researchers delivering the intervention, which may introduce expectation bias. In addition, some outcomes (such as the number of repetitions and the frequency of exercises) rely on self-reported diaries, increasing the risk of reporting bias and raising potential concerns about adherence.

Third, the aim of the study is to evaluate the combination of MI and AO therapies, and although it would also be interesting to compare the effects of this combination with those of the therapies alone, due to the difficulty in recruiting volunteers, we will not be able to conduct a study in which we can compare the combination of MI and OA and the effects of the use of MI or OA alone.

### Conclusions

The implementation of an intervention based on MI and AO has the potential to positively impact female breast cancer patients who face physical and psychological sequelae that interfere with their daily lives. This study has the potential to contribute important evidence on the effectiveness of combining MI and AO while also offering pioneering insights into the implementation of these therapies within oncology. By extending the use of these therapies to breast cancer survivorship, the findings of this study could open up new avenues for innovative, nonpharmacological strategies to improve long-term care and quality of life in this growing patient population.

## Abbreviations

ADL: activity of daily living
AO: action observation
CG: control group
CONSORT: Consolidated Standards of Reporting Trials
IG: intervention group
MI: motor imagery
NRS: numerical rating scale
SPIRIT: Standard Protocol Items: Recommendations for Interventional Trials
